# Lack of Visual Orienting to Biological Motion and Audiovisual Synchrony in 3-Year-Olds with Autism

**DOI:** 10.1371/journal.pone.0068816

**Published:** 2013-07-08

**Authors:** Terje Falck-Ytter, Erik Rehnberg, Sven Bölte

**Affiliations:** 1 Center of Neurodevelopmental Disorders at Karolinska Institutet, Department of Women’s and Children’s Health, Karolinska Institutet, Stockholm, Sweden; 2 Department of Psychology, Uppsala University, Uppsala, Sweden; Oregon Health and Science University, United States of America

## Abstract

It has been suggested that children with autism orient towards audiovisual synchrony (AVS) rather than biological motion and that the opposite pattern is to be expected in typical development. Here, we challenge this notion by showing that 3-year-old neurotypical children orient to AVS and to biological motion in point-light displays but that 3-year-old children with autism orient to neither of these types of information. Thus, our data suggest that two fundamental mechanisms are disrupted in young children with autism: one that supports orienting towards others’ movements and one that supports orienting towards multimodally specified events. These impairments may have consequences for socio-cognitive development and brain organization.

## Introduction

Orienting to biological motion is an adaptive response present early in development both in humans and in non-human animals [1], [2], [3]. In research, biological motion is typically presented in the form of point-light displays [4], showing the movement of the major joints of the body. Inversion of such point-light animations disrupts processing of biological motion [5]. If the inverted animation is presented side-by-side with an upright version, humans tend to look at the animation with the head up. Recently, Klin et al. [6] showed these inverted/upright pairs to 2-year-old children, accompanied by sound. In addition to being shown upside down, the inverted animation was also played in reverse. Consequently, the velocity profiles of the point lights in the two animations were different. This difference created different magnitudes of audiovisual synchrony (AVS; when motion changes coincide with volume changes) across the two sides of the screen.

Klin et al. [6] found that children with typical development tended to look towards the upright animation, with no indication that the spatial distribution of AVS moderated their looking patterns. Children with autism, in contrast, showed no consistent tendency to look towards the upright animation. In this group, the magnitude of AVS expressed in the upright animation correlated with the viewing preference for this animation, indicating that the children with autism oriented to AVS. Klin and Jones [7] suggested that “this pattern of looking would suggest seeing the world, and even people, as a collection of physical contingencies, unmoored from their social context or adaptive relevance” (p. 44).

These results have far-reaching implications because they point to specific processes that might be impaired in young children with autism. However, the design that Klin et al. used [6] had two limitations restricting causal inferences. First, the design included no experimental manipulation of AVS. Naturally occurring (and varying) AVS across many visually dissimilar videos was measured and related to viewing preference. Second, inversion and reversion were confounded; one animation in the pairs was always both upright and played forward in time, and the other was always both upside down and played backward in time. Consequently, any combination of these two factors could have driven any orienting response towards the upright (and forward) animation.

In addition to these methodological considerations, the finding that children with autism respond more strongly to AVS than typical children is unexpected from an empirical point of view. For example, one study that manipulated AVS experimentally found that it was a weaker orienting cue in young children with autism compared to controls [8]. In that study, a preferential looking paradigm was used, where two identical visual events were shown side-by-side, one offset from the other by three seconds. A single audio track was matched only to one of the displays. Results demonstrated that children with autism showed less preference for AVS than non-autistic children with developmental delays (matched for chronological age and functional level). The difference between autistic children and typically developing children was in the same direction and approached significance. Both of the non-autistic groups showed a robust preference for the multimodally synchronized events, but preference did not differ from chance in the autism group. The finding that typical children orient towards AVS is in line with other research suggesting that sensitivity to AVS emerges early and contributes to the child’s perception of a unified, multimodal world [9].

We designed a study similar to that of Klin et al. [6], except that in the current work, both orienting to biological motion and AVS were studied experimentally. If children with autism were to orient to AVS and ignore biological motion, that finding would support the Klin et al. hypothesis. Moreover, children with typical development should orient to biological motion and ignore AVS (by ‘ignore’, we mean that gaze performance is unaffected). According to this view, the differences between the groups of children should be particularly observable in contexts that clearly spatially separate AVS and biological motion. Thus, our study included one condition in which AVS clearly specified the inverted animation. This design also allowed us to evaluate the alternative view: Children with autism could be impaired in both orienting to AVS [8] and biological motion [10], and children with typical development could be expected to modulate looking performance as a function of changes in AVS [9], [11]. Using eye-tracking, we tested these alternative hypotheses and found clear support for the latter.

## Materials and Methods

This study was approved by the regional ethics committee of Stockholm, and we adhered to the standards specified in the 1964 Declaration of Helsinki. Written consent from caregivers was obtained.

We used non-invasive eye-tracking (Tobii T120; Tobii Technology, Stockholm, Sweden) to assess viewing preference in typically developing (n = 14; 3 females, mean/SD = 42.6/5.2 months) and autistic children (n = 10; 2 females, mean/SD = 41.0/5.3 months). Only children who had no hearing or visual impairments, no known medical conditions (e.g., epilepsy), and no neurodevelopmental disorders other than autism were included. The typical sample was recruited by summons and advertisements. Autistic children were recruited from the Autism Center for Young Children in Stockholm County and had a clinical consensus diagnosis of Autistic Disorder (hereafter ‘autism’ when referring to our sample) according to DSM-IV-TR, corroborated by information from the Autism Diagnostic Observation Schedule [12] and/or the Autism Diagnostic Interview-Revised [13]. At the Autism Center, the children received behavioral interventions ranging from intensive training to a few targeted interventions.

As in the study by Klin et al., we used the Mullen Scales of Early Learning [14] to assess developmental age. In the autism group, the developmental age (mean/SD) on the visual reception subscale was 22.7/7.7 months. On the combined language subscales, the developmental age (mean/SD) was 17.3/7.5 months. Thus, despite the fact that the present autism group was chronologically older, their developmental age was similar to the developmental age in the autistic group examined by Klin et al. [6]. The developmental age in the typical group in the present study was within the normal range (visual reception, mean/SD = 44.3/11.1 months; language subscales, mean/SD = 42.6/7.2 months). Thus, both the developmental age and the chronological age were higher in the present typical group than in the typical group included in the Klin et al. study (see also Table 1). As expected, total scores on the Social Responsiveness Scale (SRS; preschool version) [15] were higher in the autism group than in the typical group (mean/SD = 70/14 vs. 44/7, t(21) = 6.01, p<.001). To explore the role of age in typical development, we showed the main conditions (see below) also to 11 typically developing toddlers (mean/SD = 16.1/2.0 months, 4 females).

**Table 1:** Properties and findings of Klin et al. [5] and the current study.^1^

	Klin et al.	This study
Samples		
Chronological age	ASD = TD = DD (∼2 years)	ASD^2^ = TD (∼3.5 years)
Verbal function	ASD = DD; ASD<TD	ASD<TD
Nonverbal function	ASD = TD = DD	ASD<TD
*Design*		
Experimental manipulation of AVS?	No	Yes
*Main results*		
Orienting to biological motion	ASD<TD = DD	ASD<TD
Orienting to AVS	ASD>TD = DD	ASD<TD

TD = Typically Developing; DD = Developmentally Delayed; ASD = Autism Spectrum Disorder; AVS = Audiovisual Synchrony.

1 The table does not include the typically developing toddler sample from the current study.

2 The current sample included children with Autistic Disorder, only.

**Figure 1:**
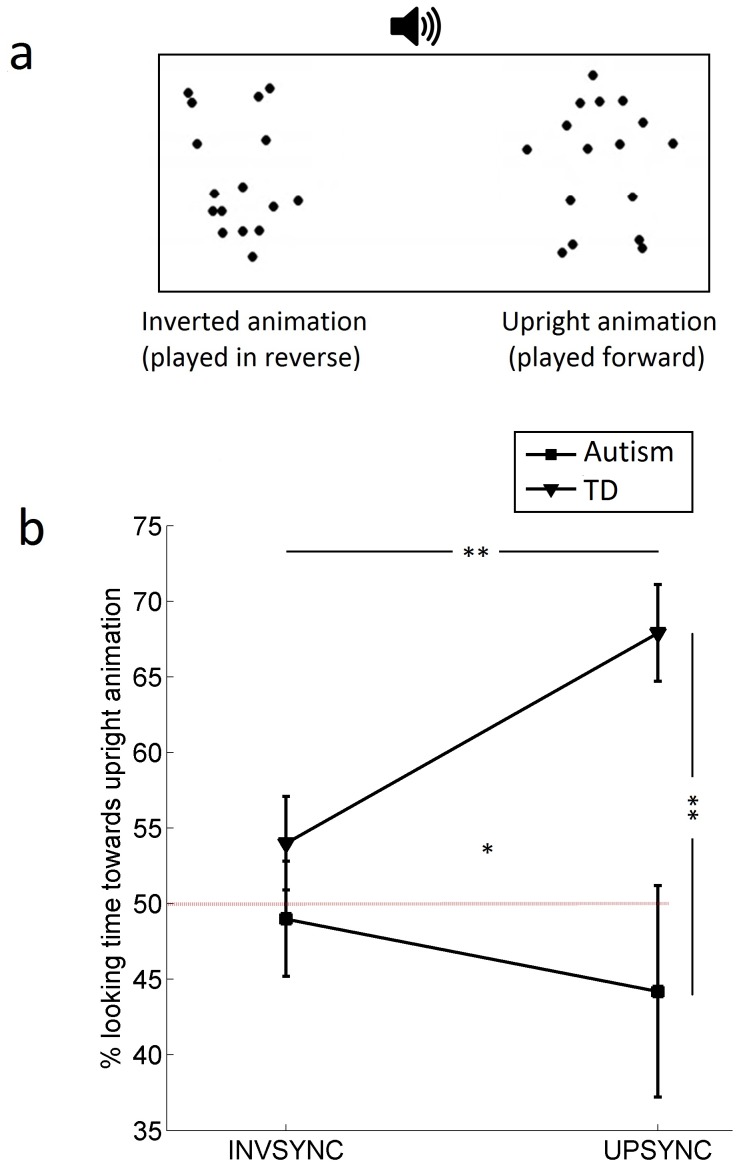
Stimuli and results. **a**) The two conditions (UPSYNC, INVSYNC) were visually identical but differed with regard to the distribution of audiovisual synchrony (AVS) across the two animations. Adopted with permission from ref. [11]. **b**) Looking preference ([upright animation]/[upright+inverted animation], in percent) was modulated by AVS only in typically developing children (TD), and AVS specifying distorted biological motion (INVSYNC condition) blocked these children’s preference for biological motion. Children with autism performed at chance level (50%) in both conditions. Error bars represent standard error of the mean; *p<.05; **p<.01.

Participants were presented several visually identical movies (30 fps) similar to Supplementary video 2 in the Klin et al. study [6]. The stimuli showed a point-light animation of a human actor (adult male) clapping hands on one side of the screen (repeatedly, large movements, ∼0.65 claps per second, 15 s duration; see Figure 1a). Simultaneously, on the other side of the screen, the same animation was shown upside down and played in reverse. As in the Klin et al. study [6], the sound of clapping hands and a human voice recording were played from a centrally placed loudspeaker. By changing the timing of the auditory clapping, AVS was manipulated to form two conditions: UPSYNC and INVSYNC (auditory clapping being synchronous with visual clapping in upright and inverted/reversed animation, respectively). Each condition included four trials, with left–right counterbalancing. We have previously presented a study where we manipulated AVS parametrically in several (five) different stimuli [11]. From these, two specific stimuli were chosen to be included in the present study because they included AVS that clearly specified either the upright or the inverted animation (here labeled UPSYNC and INVSYNC, respectively). Video S1 in the supporting information presents the UPSYNC condition with the gaze trace of one autistic child superimposed.

To produce these stimuli, we recorded human motion using a motion capture system (Qualisys, Göteborg, Sweden), which registers the position of markers placed on the joints of interest in 3D space with high temporal resolution. From these data, we created point-light movies using in-house computer software written in Matlab (MathWorks Inc., Natick, Massachusetts, USA). Video editing software (Sony Vegas; Sony Creative Software Inc., Middleton, Wisconsin, USA) was used to create the final stimuli. We selectively chose a 15-second period from the full-length motion capture movie that resulted in asynchronous visual clapping across the two animations when one of them was played backwards in time. This choice allowed manipulation of AVS by aligning auditory clapping with the visual clapping with only one of the two animations.

To isolate the effect of inversion on preferential looking [5], we also presented stimuli with one upright and one inverted animation but in which both animations were presented forwards in time (eight trials). Aside from this, the stimuli were visually identical to the UPSYNC and INVSYNC stimuli. They were accompanied by sound (social and non-social sounds, intended to test a hypothesis unrelated to the present study), but not accompanied by auditory clapping (the type of audio manipulated in UPSYNC and INVSYNC conditions). Therefore, it is likely that the absolute level of AVS was lower than in the UPSYNC and INVSYNC conditions [11]. Most important, because both animations were played forward, AVS was identical for the two animations and cannot explain preferential looking towards one of them.

The dependent variable was the proportion of looking time on the upright animation to the looking time on both upright and inverted animations (expressed as percentages). We used parametric tests (alpha level = .05) throughout unless otherwise stated. There were no outliers and no violation of the normality assumption in any group/condition. We tested the homogeneity of variance assumption for each comparison and report the adjusted statistics wherever this assumption was not met (one instance).

## Results

First, we compared the two age-matched groups. A two-way ANOVA revealed a main effect of group (F(1,22) = 12.33, p = .002, η_p_^2^ = .36), no main effect of condition (F(1,22) = 1.07, ns), and an interaction effect of group and condition (F(1,22) = 4.83, p = .039, η_p_^2^ = .18; Figure 1b). Only the typical group changed their looking performance as a function of change in AVS (t(13) = 3.23, p = .007, d >.8). The groups differed only in the UPSYNC condition (t(13.162) = 3.49, p = .007, d >.8).

Average looking time on screen was equal in the two groups (11.21 versus 11.10 s). Sticky fixations (looking at only one animation of the pair) were observed in only 2% of all trials, arguing against general attentional explanations (e.g., disengagement, shifting) [16]. Looking measures did not correlate with chronological age, developmental age, or the level of autistic traits (SRS).

When the children observed movies that played both animations forward, we found that only the typical group preferred the upright animation (t(13) = 3.67, p = .003), and the group difference was significant (t(22) = 2.18, p = .04, d >.8). Finally, the performance of the typical toddlers was strikingly similar to the performance of the older typical children (toddler data: UPSYNC mean/SD = 71%/13%; INVSYNC mean/SD = 54%/15%; t(10) = 2.501, p = .031, d >.8, paired samples t-test).

## Discussion

Klin et al. [6] suggested that AVS is a strong orienting cue for children with autism and that children with typical development ignore AVS when it is embedded in point-light displays of biological motion. We found no support for this view. Instead, the results showed that the children with autism did not orient towards AVS. Because overall looking times across the two groups were similar, the results cannot be explained by lower motivation to attend to the movies. Furthermore, we found that AVS expressed within a non-biological (inverted) animation blocked the typical children’s preference for biological motion. This outcome supports the view that AVS, like biological motion, is a strong orienting cue in typical development [8], [9], [11].

In a previous study based on the same recording of human motion and soundtracks as the present work, we found that typically developing 5-month-olds oriented towards AVS when both animations in the pairs were upright [11]. However, when we inverted one of the animations in the pairs (the one played in reverse), no such effect was identified. In this context, the infants oriented towards the upright animation regardless of our AVS manipulation. In the present study, by 16 months of age, typical infants oriented to AVS in a consistent manner even with one upright and one inverted animation in a pair. This outcome suggests that orienting responses towards AVS in this context develop between 5 and 16 months of age.

Several explanations are possible for the discrepancies between the current study and the one by Klin et al. [6]. Above all, in the present study, we manipulated AVS selectively; thus, the effect we observed cannot have been related to visual properties per se because the two conditions were visually identical. In the Klin et al. study [6], the stimuli were not visually identical. It should be noted also that although the sound was manipulated (the timing of auditory clapping), it was played from a centrally placed loudspeaker. Thus, the preference change for our manipulation cannot be attributable to auditory properties per se either (this factor was also controlled in Klin et al. [6]).

The type of AVS investigated in the present study was similar to the type of AVS found to be a strong orienting cue in children with autism in the Klin et al. studies [6], [7]. The most direct comparison can be done for the current UPSYNC condition, which is analogous to Supplementary Video 2 in the Klin et al. study [6], where auditory clapping occurred in synchrony with visual clapping in the upright animation. In both the current UPSYNC condition and the Supplementary video 2 in Klin et al. [6], AVS clearly specified the upright animation. However, although Klin et al. [6] found that children with autism oriented to the upright animation in this particular context, the children with autism in the present study did not.

The INVSYNC condition, in which AVS primarily specified the inverted animation [11], has no analogue in the Klin et al. study [6]. However, in the Klin et al. study, the relationship between AVS and orienting was linearly modeled across ‘human voice only’ as well as ‘human voice plus clapping’ soundtracks. Thus, according to this model, the current INVSYNC condition would be expected to cause the most extreme group difference. That is, in the INVSYNC condition, children with autism would be expected to look towards the inverted animation (because it contained the most AVS), and children with typical development would be expected to look towards the upright animation (because they orient towards biological motion and ignore AVS). This prediction was not supported by our results. Finally, it is worth noting that Klin et al. used animations that lasted 30 seconds on average whereas the present study used animations lasting 15 seconds.

Another important distinction is the characteristics of the autistic samples included in the two studies (Table 1). First, our autism sample consisted of children with an Autistic Disorder diagnosis only. The Klin et al. [6] study included children with Autistic Disorder (the majority) and Autism Spectrum Disorder (ASD) diagnoses. Second, in the Klin et al. study [6], the autistic children did not differ from the control groups in terms of non-verbal mental age (or chronological age). In contrast, our autism group was developmentally delayed (which is not unexpected; e.g. Kim and Lord [17] reported that in a large sample of children ages 21 to 47 months of age with autism spectrum disorders, the majority were non-verbal and had non-verbal IQ scores two to three SDs below what is expected in typical development). Because the present autism group’s developmental age was similar to the developmental age of the autistic children in the Klin et al. [6] study, differences in developmental age are not a likely explanation for the discrepancy between the studies in terms of autism group results. However, the autistic children in our study were about one chronological year older than those in Klin et al. [6]. Thus, the difference between the studies could reflect that preference for AVS diminishes from the second to the third year of life in autistic children, although the lack of correlation with age in our study speaks against this interpretation. It is also conceivable that the association between AVS and viewing in children with autism is moderated by a combination of factors related to chronological and developmental age. Future investigations should relate orienting to AVS to these variables systematically. However, our results suggest that orienting to AVS rather than biological motion is not a valid generic description of how children with autism perform in this context. Moreover, the results from the two normally developing groups included in the current study indicate that orienting towards AVS in this context is not an atypical response.

Regarding processing of biological motion, the picture is much clearer. In the videos where the level of AVS was equalized across the two animations, the typical children looked preferentially at the upright animation while the autism group did not. The group difference was significant. Several studies (including the current one), using different types of control stimuli, now agree that young children with autism orient less to biological motion than typically developing children [6], [10], [18]. Whether biological motion processing is different in infants who later receive an autism diagnosis is an interesting question for further research [19].

A limitation of the present study is its small sample size. Replication in further studies and across different contexts (e.g., different types of biological motion stimuli) is needed before firm conclusions can be drawn. Nevertheless, our findings suggest that two adaptive mechanisms may be disrupted in 2-to-4-year-old children with autism: one supporting orientation towards biological motion and one supporting orientation towards multimodally specified events [8], [10]. In addition to their theoretical relevance, these findings may have clinical implications. For example, early intervention strategies could differ dependent on whether the key problem is hyposensitivity to biological motion or hypersensitivity to AVS. Of interest, both mechanisms map onto a broader theme of impaired binding of information (visuospatially and across modalities), which could potentially be linked to altered patterns of neural connectivity found in autism [20]. On a psychological level, the results may indicate that children with autism perceive people and events as disparate, unimodal phenomena, rather than as multimodally unified entities [6], [7]. Such a fragmented perception early in life is likely to have negative consequences for socio-cognitive development and brain organization [21].

## Supporting Information

Video S1 **This video presents the UPSYNC condition with the gaze trace of one autistic child superimposed.**
(AVI)
